# The Glycosylation of Serum IgG Antibodies in Post-COVID-19 and Post-Vaccination Patients

**DOI:** 10.3390/ijms26020807

**Published:** 2025-01-18

**Authors:** Csaba Váradi

**Affiliations:** Institute of Chemistry, Faculty of Materials and Chemical Engineering, University of Miskolc, 3515 Miskolc, Hungary; csaba.varadi@uni-miskolc.hu; Tel.: +30-894-7730

**Keywords:** antibody glycosylation, COVID-19, vaccination, liquid chromatography

## Abstract

The signature of human serum IgG glycosylation is critical in the defense against pathogens. Alterations of IgG N-glycome were associated with COVID-19 (Coronavirus disease 2019) severity, although knowledge on the response to vaccination is limited. IgG N-glycome was analyzed in this study in post-COVID-19 and post-vaccination patients to reveal potential glycosylation-based alterations using hydrophilic interaction liquid chromatography (HILIC-UPLC) with fluorescence (FLR) and mass-spectrometric (MS) detection. IgG antibodies were purified from serum samples through protein G affinity chromatography followed by PNGase F digestion-based deglycosylation. The released glycans were fluorescently derivatized by procainamide labeling and purified via solid-phase extraction. Higher levels of sialylation and afucosylation were identified in post-COVID-19 patients, which was further expanded by vaccination, but only in those who were previously SARS-CoV-2 (Severe acute respiratory syndrome coronavirus 2) infected.

## 1. Introduction

Circulating antibodies in the blood are one of the most important defensive attributes of the immune system during bacterial and viral infections [1]. Serum antibodies are glycoproteins with five major classes based on their heavy chains (IgG, IgM, IgA, IgD, and IgE), which serve as a protective barrier against pathogens [2]. During viral infection, an increased IgM level is the primary immune response indicating the production of IgG antibodies with high affinity to the antigen as a secondary response [3]. The most dominant immunoglobulin class in human serum is IgG, which consists of two heavy and two light chains connected by disulfide bridges, while functionally, they are composed of a variable (Fab)2 and a constant Fc domain [4]. The (Fab)2 domain is responsible for the neutralization of the antigens, while the Fc region coordinates the Fc receptors and complement proteins [5]. IgG antibodies are glycoproteins with a conserved glycosylation site at Asn-297 of the heavy chain, representing mainly bi-antennary glycans with α1-6-linked core-fucose [6]. Glycosylation is a critical quality attribute of IgGs with an evident role in the protection against bacterial and viral infections [7]. Glycans influence the stability, half-life, conformation, and activity of IgG antibodies but also effect on their pro- or anti-inflammatory status [8]. In the last decade, the glycobiology of IgG has become an explosive research area due to its high-throughput separation and high-resolution detection techniques [9]. IgG N-glycome was associated with various pathological states including inflammatory and malignant diseases as well as pregnancy, aging, and smoking [10]. The importance of terminal monosaccharides has been described in relation to the effector functions of IgGs, such as sialylation in anti-inflammatory activity, galactosylation in complement activation, and fucosylation in antibody-dependent cellular cytotoxicity [11]. IgG glycosylation has received special attention during the coronavirus pandemic [12]. Signatures of IgG glycosylation were found to correlate with the severity of coronavirus disease 2019 (COVID-19) and vaccine antibody response [13]. Changes in IgG N-glycome were observed during the course of COVID-19 [14]. The unique glycosylation pattern of serum IgGs after BNT162b2 mRNA vaccination has also been described [15]. In this study, IgG glycosylation was analyzed in patients after SARS-CoV-2 infection and after mRNA vaccination in order to identify potential glycosylation-based alterations using hydrophilic interaction liquid chromatography with fluorescence detection. IgG antibodies were purified from serum samples through protein G affinity chromatography followed by PNGase F digestion-based deglycosylation. The released glycans were fluorescently derivatized via procainamide labeling and purified using solid-phase extraction. The prepared samples were analyzed with HILIC-UPLC and quantified in UNIFI based on the fluorescence spectra with MS confirmation. Multiple statistical tests were performed in order to identify potential alterations in the IgG N-glycome. The aim of this study was to investigate the differences in IgG N-glycosylation in patients after COVID-19 infection and/or mRNA vaccination.

## 2. Results

**Figure 1:**
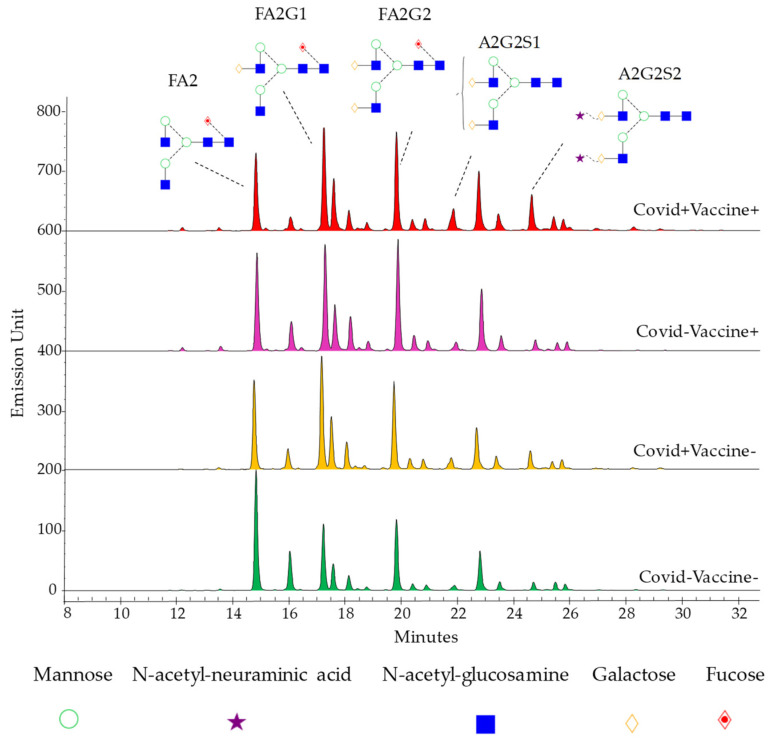
Fluorescence chromatograms of serum IgG N-glycome of Covid−Vaccine−, Covid+Vaccine−, Covid−Vaccine+, and Covid+Vaccine+ patients via UPLC-HILIC-FLR (main structures highlighted; FA2: fucosylated bi-antennary; FA2G1: fucosylated and mono-galactosylated bi-antennary; FA2G2: fucosylated and bi-galactosylated bi-antennary; A2G2S1: mono-sialylated and bi-galactosylated bi-antennary; A2G2S2: bi-sialylated and bi-galactosylated bi-antennary).

A total of 16 Covid−Vaccine−, 16 Covid+Vaccine−, 16 Covid−Vaccine+, and Covid+Vaccine+ patient samples were analyzed using HILIC-FLR-MS in triplicate. Representative chromatograms are shown in Figure 1, with the main structures identified. The relative quantitation of the individual glycan structures was performed using fluorescence chromatograms, where 19 peaks were integrated with MS confirmation. The identified components and corresponding details including retention time and observed *m/z* values are listed in Supplementary Table S1.

**Figure 2:**
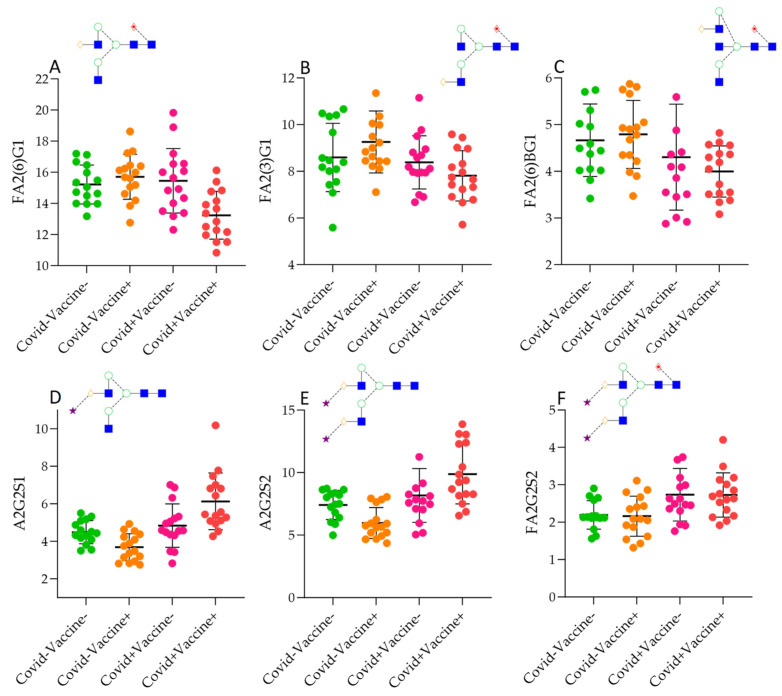
Significantly different IgG N-glycan ratios in Covid−Vaccine−, Covid+Vaccine−, Covid−Vaccine+, and Covid+Vaccine+ patients. (**A**): FA2(6)G1 (bi-antennary fucosylated mono-galyactosylated on the upper arm, (**B**): FA2(3)G1 (bi-antennary fucosylated mono-galyactosylated on the lower arm, (**C**): FA2B(6)G1 (bi-antennary fucosylated mono-galyactosylated with a bisecting N-acetyl-glucosamine, (**D**): A2G2S1: bi-antennary monosialylated, (**E**): bi-antennary bi-sialylated, (**F**): bi-antennary bi-sialylated core-fucosylated.

The generated data were used for statistical tests to find correlation and significant differences between the four patient groups. In the case of the 12 structures, significantly different ratios were detected through the Kruskall–Wallis test, as shown in Supplementary Table S2. Most of the identified significances were obtained between the Covid−Vaccine− and Covid+Vaccine+ groups, with the trend being a lower level of neutral structures (Figure 2A–C) in Covid+Vaccine+ patients and a higher ratio of sialylated glycans (Figure 2D–F). This trend was similar in the Covid+Vaccine−, group while in the Covid−Vaccine+ patients, a lower level of sialylation was found compared to the Covid−Vaccine− group. Using the Mann–Whitney pairwise comparison, significant differences were revealed in Covid−Vaccine+ patients compared to Covid−Vaccine− patients, namely, higher FA2G2 and lower A2G2S2 levels. Interestingly, in the Covid+Vaccine− group, the lower FA2B(3)G1 and higher FA2G2S2 levels were significant (Supplementary Table S2). Despite of the lower sialylation in Covid−Vaccine− patients, in the Covid+ groups, a similar pattern was identified in response to vaccination rather than in response to COVID-19 positivity. As shown in Supplementary Table S2, in Covid+Vaccine+ patients, significantly higher levels of A2G2S1 and A2G2S2 were detected even after Bonferroni correction, suggesting higher levels of afucosylation and sialylation compared to the Covid−Vaccine+ group. Overall, vaccination resulted in lower sialylation in the Covid− groups, while it was significantly higher in Covid+ patients, but mainly on non-fucosylated structures, while COVID-19 positivity resulted in higher sialylation and afucosylation in both cases.

**Figure 3:**
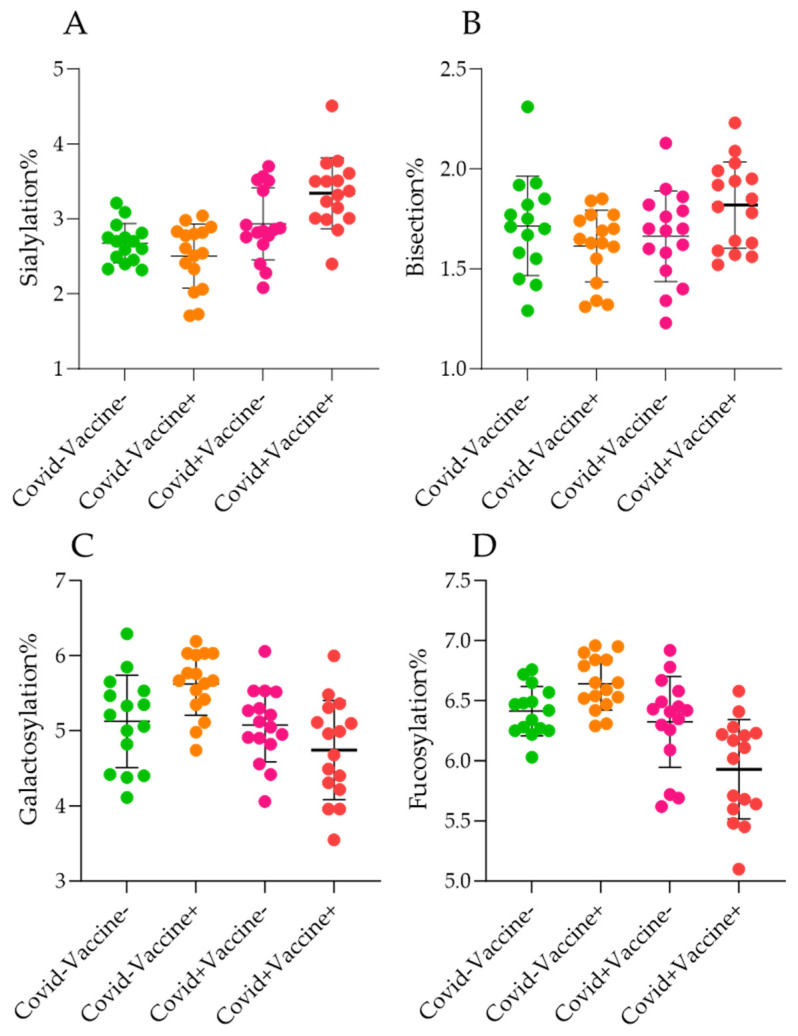
The level of sialylation (**A**), bisection (**B**), galactosylation (**C**), and fucosylation (**D**) in Covid−Vaccine−, Covid+Vaccine−, Covid−Vaccine+, and Covid+Vaccine+ patients.

The importance of terminal monosaccharide units of IgG glycans has been described regarding the effector functions and half-life of the antibody [11]. As shown in Figure 3, we examined the level of different glycan clusters in the analyzed patient groups. Similarly to the Kruskall–Wallis test on Figure 2, a higher level of total sialylation was found in Covid+Vaccine− and Covid+Vaccine+ patients. Interestingly, vaccination resulted in higher sialylation only in the post-COVID-19 patients, while COVID-19 positivity indicated a higher level of sialic acid containing glycans in each case. This has crucial importance as sialylation is often associated with the anti-inflammatory activity of IgGs, as terminal sialylation negatively affects antibody binding to the FcγRIIIa receptor [11]. As shown in Figure 3B, the level of bisecting N-acetyl-glucosamine-containing glycans was almost in the same range across the examined groups, while galactosylation (Figure 3C) and fucosylation (Figure 3D) showed similar tendencies. Both galactosylation and fucosylation levels were found to be slightly higher in the Covid−Vaccine+ group than in Covid−Vaccine− patients, while lower levels were detected in the Covid+Vaccine− and Covid+Vaccine+ groups. As can be seen in Figure 3, the most prominent differences were the higher sialylation and lower galactosylation as well as fucosylation in post-COVID-19 patients.

**Figure 4:**
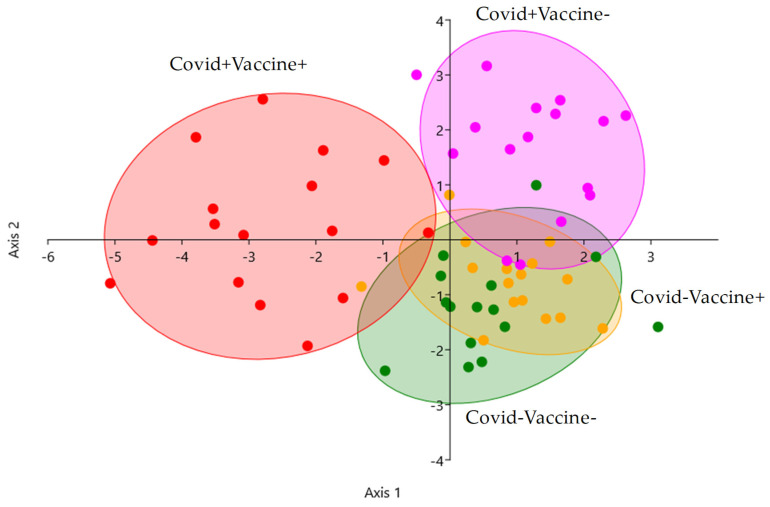
Linear discriminant analysis of Covid−Vaccine− (green), Covid+Vaccine− (purple), Covid−Vaccine+ (orange), and Covid+Vaccine+ (red) patients based on their IgG N-glycome.

Linear discriminant analysis was performed to visualize the possibility of separating the patient groups based on their IgG glycosylation pattern. As it shown in Figure 4, post-COVID-19 patients were well separated, and the effect of vaccination was also detectable, as the Covid+Vaccine+ group was perfectly separated. The Covid+Vaccine− group can also be distinguished from the Covid−Vaccine− and Covid−Vaccine+ groups, while the Covid− patients overlapped, as no alterations were identified in response to vaccination in Covid−Vaccine+ patients. Our results suggest that IgG antibodies have a higher level of sialylation in post-COVID-19 patients than post-vaccination patients, although after COVID-19 positivity, vaccination can further increase the number of sialylated and afucosylated IgGs.

## 3. Discussion

The main limitation of this study is the low number of analyzed samples narrowing down the possibility of a comprehensive discussion, although some of the previous reports described similar findings. Hou et al. reported lower fucosylation in COVID-19 patients, while the abundance of sialylated glycans was higher and the sialylation was in correlation with the severity of the disease [16]. The importance of lower fucosylation was described by Chakraborty et al. in COVID-19 patients, where the afucosylated antibody responses were associated with disease severity [17]. They also claimed that SARS-CoV-2 infection and mRNA vaccination both elicited high neutralizing titers of afucosylated and sialylated IgGs. Beimdiek et al. reported increased levels of sialylated bi-antennary and decreased levels of neutral glycans in COVID-19 patients compared to controls [18]. All these previous reports support the findings of this current study, as galactosylation and fucosylation were found to be lower in post-COVID-19 and post-vaccination patients.

## 4. Materials and Methods

### 4.1. Chemicals

Formic acid, ammonium hydroxide, acetic acid, acetonitrile, picoline borane, procainamide–hydrochloride, and dimethyl sulfoxide were obtained from Sigma-Aldrich (St. Louis, MO, USA). PNGase F was ordered from New England Biolabs (Ipswich, MA, USA).

### 4.2. Patient Samples

Serum samples from 64 patients (16 Covid−Vaccine−, 16 Covid−Vaccine+, 16 Covid+Vaccine−, and 16 Covid+Vaccine+) were collected at the Borsod Academic County Hospital (Miskolc, Hungary). Baseline characteristics of the collected patient samples are summarized in Supplementary Table S3. Serum samples were obtained 1 month after the injection of the second dose of the Pfizer–BioNTech COVID-19 mRNA vaccine. The study was approved by the Regional Research Ethics Committee (ethical approval number: BORS-02-2021). Informed consent forms were signed by each patient in accordance with the Declaration of Helsinki.

### 4.3. N-Glycan Release from Serum Proteins, Labeling, and Clean-Up

The glycan release was conducted following the New England Biolabs (Ipswich, MA, USA) PNGase F deglycosylation protocol using 9 µL of serum sample. The liberated carbohydrates were fluorescently labeled through the addition of 10 μL 0.3 M procainamide and 300 mM picoline borane in 70%/30% of dimethyl sulfoxide/acetic acid, incubating for 4 h at 65 °C. The purification of labeled glycans was performed through NH_2_-functionalized MonoSpin columns (GL Sciences Inc., Tokyo, Japan) according to the manufacturer’s protocol. The purified glycans were dissolved in 25%/75% water/acetonitrile and analyzed by HILIC-UPLC-FLR-MS.

### 4.4. UPLC-FLR-MS Analysis

The fluorescently labeled N-glycans were analyzed using a Waters Acquity ultra-performance liquid chromatography system equipped with a fluorescence detector and a Xevo-G2S qTOF mass spectrometer under the control of MassLynx 4.2 (Waters, Milford, MA, USA). Separations were performed using a Waters BEH Glycan column, 100 × 2.1 mm i.d., 1.7 μm particles, with a linear gradient of 75–55% acetonitrile (Buffer B) at 0.4 mL/min in 22 min, using 50 mM ammonium formate pH 4.4 as Buffer A. Then, 1 μL of sample was injected in all runs while the sample manager temperature was 15 °C and the column temperature was 60 °C. The fluorescence detection excitation and emission wavelengths were λ_ex_ = 308 nm and λ_em_ = 359 nm. In the MS analysis, 3 kV electrospray voltage was applied to the capillary. The desolvation temperature was 120 °C and the desolvation gas flow was 800 L/h. Mass spectra were acquired using positive-ionization mode over the range of 500–3000 *m*/*z.*

### 4.5. Data Analysis

The chromatograms of the analyzed patient samples were integrated in Unifi chromatography software 3.0.0.15 (Waters, Milford, MA, USA) based on the fluorescence spectra with MS confirmation. The mass-to-charge ratio of the individual glycan structures was determined in GlycoWorkbench 2.0. The statistical analyzes were performed in IBM SPSS Statistics 23 to perform the Kruskal–Wallis test and Mann–Whitney pairwise comparison. Linear discriminant analysis was carried out in Past 4.11 software. The figures were created in GraphPad prism 10.

## 5. Conclusions

IgG glycosylation was analyzed in this study in post-COVID-19 and post-vaccination patients using HILIC-UPLC with fluorescence and mass-spectrometric detection. Higher sialylation and lower fucosylation were identified in response to COVID-19 infection, which was similar after vaccination, but only in post-COVID-19 patients. Our results suggest that COVID-19 infection might elicit a more powerful immune response than vaccination, although the use of vaccination can still enhance the level of highly sialylated and afucosylated IgGs.

## Data Availability

The generated data can be requested from the corresponding author.

## Supplementary Materials

The following supporting information can be downloaded at: https://www.mdpi.com/article/10.3390/ijms26020807/s1.

## References

[1] Nicholson L.B. (2016). The immune system. Essays Biochem..

[2] Vidarsson G., Dekkers G., Rispens T. (2014). IgG subclasses and allotypes: From structure to effector functions. Front. Immunol..

[3] Murin C.D., Wilson I.A., Ward A.B. (2019). Antibody responses to viral infections: A structural perspective across three different enveloped viruses. Nat. Microbiol..

[4] Schroeder H.W., Cavacini L. (2010). Structure and function of immunoglobulins. J. Allergy Clin. Immunol..

[5] Zhao J., Nussinov R. (2019). Antigen binding allosterically promotes Fc receptor recognition. MAbs.

[6] Krištić J., Lauc G. (2024). The importance of IgG glycosylation—What did we learn after analyzing over 100,000 individuals. Immunol. Rev..

[7] Irvine E.B., Alter G. (2020). Understanding the role of antibody glycosylation through the lens of severe viral and bacterial diseases. Glycobiology.

[8] Boune S., Hu P., Epstein A.L., Khawli L.A. (2020). Principles of N-Linked Glycosylation Variations of IgG-Based Therapeutics: Pharmacokinetic and Functional Considerations. Antibodies.

[9] Shkunnikova S., Mijakovac A., Sironic L., Hanic M., Lauc G., Kavur M.M. (2023). IgG glycans in health and disease: Prediction, intervention, prognosis, and therapy. Biotechnol. Adv..

[10] Reily C., Stewart T.J., Renfrow M.B., Novak J. (2019). Glycosylation in health and disease. Nat. Rev. Nephrol..

[11] Raju T.S. (2008). Terminal sugars of Fc glycans influence antibody effector functions of IgGs. Curr. Opin. Immunol..

[12] Pongracz T., Vidarsson G. (2022). Antibody glycosylation in COVID-19. Glycoconj. J..

[13] Ash M.K., Bhimalli P.P., Cho B.K., Mattamana B.B., Gambut S., Tarhoni I., Fhied C.L., Reyes A.F., Welninski S.J., Arivalagan J. (2022). Bulk IgG glycosylation predicts COVID-19 severity and vaccine antibody response. Cell Rep..

[14] Petrović T., Vijay A., Vučković F., Trbojević-Akmačić I., Ollivere B.J., Marjanović D., Bego T., Prnjavorac B., Đerek L., Markotić A. (2022). IgG N-glycome changes during the course of severe COVID-19: An observational study. EBioMedicine.

[15] Farkash I., Feferman T., Cohen-Saban N., Avraham Y., Morgenstern D., Mayuni G., Barth N., Lustig Y., Miller L., Shouval D.S. (2021). Anti-SARS-CoV-2 antibodies elicited by COVID-19 mRNA vaccine exhibit a unique glycosylation pattern. Cell Rep..

[16] Hou H., Yang H., Liu P., Huang C., Wang M., Li Y., Zhu M., Wang J., Xu Y., Wang Y. (2021). Profile of Immunoglobulin G N-Glycome in COVID-19 Patients: A Case-Control Study. Front. Immunol..

[17] Chakraborty S., Gonzalez J.C. (2022). Early non-neutralizing, afucosylated antibody responses are associated with COVID-19 severity. Sci. Transl. Med..

[18] Beimdiek J., Janciauskiene S., Wrenger S., Volland S., Rozy A., Fuge J., Olejnicka B., Pink I., Illig T., Popov A. (2022). Plasma markers of COVID-19 severity: A pilot study. Respir. Res..

