# Supplementary material for: The Glycosylation of Serum IgG Antibodies in Post-COVID-19 and Post-Vaccination Patients
Source: Int J Mol Sci. 2025 Jan 18;26(2):807. doi: 10.3390/ijms26020807 (PMC11765615; doi:10.3390/ijms26020807)
Supplement: Supplementary file 1 [file ijms-26-00807-s001.zip › ijms-3384946-supplementary.pdf]

Supplementary

The Glycosylation of Serum IgG Antibodies in post-Covid-19 and post-Vaccinated Patients

Csaba Váradi *

Institute of Chemistry, Faculty of Materials Science and Engineering, University of Miskolc, Hungary 3515
* Correspondence: csaba.varadi@uni-miskolc.hu; Tel.: +30-894-7730 (Cs.V.)

Supplementary Table S1. The identified glycan structures in Unifi based on their retention time and mass to charge ratio (m/z)

**Component name**	**Neutral mass (Da)**	**Observed m/z**	**Mass error (ppm)**	**Observed RT (min)**	**Adducts**
A2	1316,487	1536,664	-2,5	13,77	PA_H, PA_2H
FA2	1462,544	841,8638	-2,9	15,07	PA_2H, PA_H
FA2B	1665,624	943,4028	-3,3	16,38	PA_2H, PA_H
FA2(6)G1	1624,597	922,8898	-3,1	17,61	PA_2H, PA_H
FA2(3)G1	1624,597	922,8892	-3,8	17,98	PA_2H, PA_H
FA2B(6)G1	1827,677	1024,428	-4,6	18,61	PA_2H
FA2B(3)G1	1827,677	1024,427	-5,6	18,95	PA_2H
A2G2	1640,592	930,8857	-4,7	19,24	PA_2H, PA_H
FA2G2	1786,65	1003,916	-3,4	20,35	PA_2H
FA2BG2	1989,729	1105,453	-5,2	21,01	PA_2H
FA2G1S1	1915,693	1068,434	-5,7	21,2	PA_2H
A2G2S1	1931,688	1076,433	-4,9	22,29	PA_2H
FA2G2S1	2077,746	1149,461	-4,8	23,25	PA_2H
FA2BG2S1	2280,825	1251	-4,9	24,05	PA_2H

A2G2S2	2222,783	1221,979	-5,1	25,11	PA_2H
FA2G2S2	2368,841	1295,007	-5,9	25,95	PA_2H

Supplementary Table S2. Significant differences of Kruskall-Wallis test and Bonferroni corrected Mann-Whitney pairwise comparisons

	Kruskall-Wallis	Mann-Whitney				
		Covid-Vaccine-vs Covid-Vaccine+	Covid-Vaccine- vs Covid+Vaccine-	Covid-Vaccine- vs Covid+Vaccine+	Covid-Vaccine+ vs Covid+Vaccine+	Covid+Vaccine- vs Covid+Vaccine+
A2	0,390	0,338	0,140	0,175	0,445	0,590
FA2	0,152	0,281	0,470	**0,041**	0,381	0,080
FA2B	0,390	0,264	0,151	0,140	0,642	0,809
FA2(6)G1	**0,001**	1	1	**0,001**	**0,001**	**0,011**
FA2(3)G1	**0,017**	1	1	0,051	**0,01**	1
FA2B(6)G1	**0,038**	1	1	**0,201**	**0,06**	1
FA2B(3)G1	0,129	0,401	**0,037**	0,054	0,287	0,985
A2G2	0,266	0,495	0,358	0,202	0,423	**0,032**
FA2G2	**0,040**	**0,13**	1	1	**0,068**	1
FA2BG2	0,319	0,188	0,626	0,572	0,445	0,305
FA2G1S1	0,914	0,682	0,892	0,599	0,926	0,539
A2G2S1	**0,191**	**0,005**	1	**0,000**	**0,000**	**0,103**
FA2G2S1	0,542	0,188	0,379	0,470	0,539	0,867
FA2BG2S1	0,068	0,446	0,423	0,060	**0,014**	0,341
A2G2S2	**0,000**	**0,191**	1	**0,014**	**0,000**	**0,103**
FA2G2S2	**0,008**	1	**0,196**	**0,1**	**0,044**	1

Supplementary Table S3. Baseline characteristics of the collected patient samples

	Covid-Vaccine- Average	std	Covid-Vaccine+ Average	std	Covid+Vaccine- Average	std	Covid+Vaccine+ Average	std
**Age**	46,56	9,51	46,13	10,05	45,19	9,52	42,81	11,69
**C reactive protein**	4,09	5,11	1,50	1,72	1,72	2,21	3,64	3,80
**White blood cell**	7,67	2,04	7,51	2,48	7,53	1,68	6,97	1,51
**Hemoglobin**	135,94	14,36	133,88	13,68	172,00	109,35	142,13	11,25
**Thrombocyte**	270,50	63,31	301,00	65,01	280,07	80,63	298,19	57,33
**Neutrophil**	4,51	1,09	4,66	1,89	37,99	28,20	4,04	1,10
**lymphocyte**	2,33	0,93	2,15	0,70	18,54	13,91	2,23	0,48
**eosinophyl**	0,15	0,08	0,09	0,06	1,23	1,34	0,14	0,07
**Vitamin D3**	91,84	49,89	89,88	36,94	96,98	42,72	66,64	29,42
